# Detection of antibodies against hepatitis E virus in pet veterinarians and pet dogs in South Korea

**DOI:** 10.1186/s13620-019-0146-4

**Published:** 2019-07-22

**Authors:** Kwang-Soo Lyoo, Soo-Jin Yang, Woonsung Na, Daesub Song

**Affiliations:** 1 Korea Zoonosis Research Institute, Chonbuk National University, Iksan, South Korea; 2 School of Bioresources and Bioscience, Chung-Ang University, Anseong, South Korea; 3 College of Veterinary Medicine, Chonnam National University, Gwangju, South Korea; 4 College of Pharmacy, Korea University, Sejong, South Korea

**Keywords:** Hepatitis E virus, Pet dogs, Pet veterinarians, South Korea

## Abstract

Hepatitis E virus (HEV) is a zoonotic pathogen commonly considered an important foodborne virus. Pet dogs are important reservoirs of zoonotic agents. In the present study, the seroprevalence of HEV in pet dogs and pet veterinarians were found to be 28.2 and 5.0%, respectively. It remains unclear whether pet veterinarians are at higher risk of HEV transmission. However, pet animals and individuals who have contact with infected animals must be continually monitored for public health concerns.

## Introduction

Hepatitis E virus (HEV) is commonly considered an important foodborne virus [1]. From a public health perspective, the identification and contamination of HEV in food animals and meat products have been associated with the risk of zoonotic infection and food safety [2, 3]. HEV belongs to the genus *Hepevirus* in the family *Hepeviridae*, which is a small non-enveloped, single-strand RNA virus [4]. There are four HEV genotypes: genotype-1 and genotype-2 cause the endemic outbreak of hepatitis E in humans in developing countries, whereas genotype-3 and genotype-4 are zoonotic agents associated with sporadic infections in humans, domestic animals, and wild animals worldwide [1, 4]. Particularly, pigs have been considered potential reservoir species of HEV because swine HEV showed not only high genetic homology with human HEV but also interspecies transmission to humans was experimentally demonstrated in non-human primates [3, 5].

Antibodies against HEV were first identified in dogs in India in 2001 [6]. Subsequently, HEV seroprevalence in dogs has been observed in the UK, Germany, and China [7–9]. Due to public health issues, pet animals have been eventually considered important reservoirs of zoonotic agents owing to their close contact with humans and other pets [10]. Indeed, recent seroprevalence studies have shown that a higher seropositivity rate of anti-HEV IgG was detected in veterinarians than in the general population [11, 12]. They suggested that occupational contact with infected animals may be associated with the risk of zoonotic HEV transmission, and veterinarians potentially have an increased risk of exposure and infection caused by this virus [11, 12]. Therefore, we aimed to investigate the presence of HEV antibodies in pet dogs in South Korea. Additionally, the antibodies were tested using sera collected from pet veterinarians to validate the zoonotic risk of HEV in South Korea.

## Materials and methods

Serum samples from dogs were randomly collected from small animal clinics in Seoul, Gyeonggi, and Jeonbuk provinces in South Korea. The sampling followed the General Animal Care Guideline as required and approved by the Institutional Animal Care and Use Committee of Chonbuk National University (approval no.: CBNU-2018-063). A total of 287 serum samples were collected from pet dogs with or without gastroenteritis symptoms, such as diarrhea or vomiting, and were stored at − 20 °C until transportation to the laboratory. Pet veterinarians were asked to volunteer for this study, and serum samples were collected by convenience sampling from 40 participants working in companion animal clinics in Seoul, Gyeonggi, and Jeonbuk provinces in South Korea. This study was approved and performed according to the guidelines of the Institutional Review Board of Chonbuk National University (IRB no.: CBNU-2018-09-003-001). All sera were stored at − 20 °C until transportation to the laboratory.

Anti-HEV IgG antibodies were detected using a commercial ELISA kit (Wantai Biological Pharmacy Co, Beijing, China) according to the manufacturer’s instructions. Briefly, 100 μl of dilution buffer was added into microplate wells, then 10 μl of serum sample was added, gently mixed, and incubated at 37 °C for 30 min. After washing, 100 μl of horseradish peroxidase conjugate was added and incubated for 30 min and washed. One-hundred microliters of tetramethylbenzidine substrate was added and incubated at 37 °C for 15 min, then the reaction was stopped. Optical density (OD) was read at 450 nm wavelength using a microplate reader (Model 680) (Bio-Rad, CA, USA), and the cutoff value was calculated as 0.16 plus mean OD value of the negative control based on maximizing true positives and minimizing false negatives. All sera, including a negative control serum, were tested in duplicate. Statistical analyses of seropositivity against HEV in dogs with or without gastroenteritis symptoms were performed using the Fisher’s exact test. Values of *p* < 0.05 were considered statistically significant.

## Results and discussion

Serum samples obtained from pet dogs were divided into two groups according to the presence of gastroenteritis symptoms, such as diarrhea or vomiting. Among the 287 dog serum samples collected from South Korea, the seropositivity rate of the gastroenteritis symptom and non-gastroenteritis symptom groups was 30.7% (16/52) and 27.6% (65/235), respectively. The seropositive rate of both groups was not significantly different (*p* > 0.73, odds ratio: 1.16, 95% confidence interval: 0.60–2.24). Moreover, approximately 5% (2/40) of pet veterinarians tested positive for anti-HEV IgG (Table 1).

**Table 1:** The seroprevalence of anti-HEV antibody in pet veterinarians and pet dogs

	Pet veterinarians	Dogs		
		Gastroenteritis symptoms	Non-gastroenteritis symptoms	Total
Samples	40	52	235	287
Seropositive	2	16	65	81
Rates	5.0%	30.7%	27.6%	28.2%

Optical density (OD) values of positive samples ranged from 0.33 to 1.26. Negative sera OD values were less than 0.08

Pet dogs are commonly considered a family member, and they have intimate contact with humans in most countries. Hence, zoonotic transmission of foodborne pathogens could be through direct contact with infected dogs or indirect contact with objects, such as contaminated food. In fact, the transmission of foodborne bacteria, such as *Escherichia coli,* and *Staphylococcus aureus*, between dogs and humans has been observed in some cases [13, 14]. Moreover, pet dogs were considered potential reservoirs of the human norovirus because a strain identical to that from a patient with norovirus infection was detected in stool samples of the patient’s pet dogs [15]. Therefore, it may be worthwhile to investigate the zoonotic risk of HEV in dogs for public health concern.

In this study, all serum samples from pet dogs were collected from small animal clinics in the metropolitan area, and the overall seropositivity rate was 28%, which is similar to that observed in previous studies showing that seropositivity to HEV among pet dogs ranged from 19 to 30% in urbanized provinces in China [11]. Although the clinical signs of HEV infection in dogs have not yet been demonstrated, we compared the seropositivity rate between both groups of dogs with or without gastroenteritis symptoms. Consequently, the difference in seropositivity to HEV between both groups was statistically insignificant.

To date, only two studies have investigated seroprevalence in pet veterinarians [10, 12]. In a preceding study, the rate of HEV seropositivity was not significantly different between pet veterinarians (9.7%) and the general population (13.3%) in Portugal, and it was suggested that the veterinarians have no increased risk to HEV infection [10]. Conversely, the other study revealed that the rate of HEV seropositivity among pet veterinarians (17.8%) was significantly higher than that of non-veterinarians (5.8%) in Finland [12]. In the present study, the rate of HEV seropositivity among pet veterinarians in South Korea was 5.0%, whereas that the general population was 5.9% according to a previous nationwide survey in South Korea [16]. Therefore, it could be regarded that HEV seropositivity among pet veterinarians and the general population may not be significantly different in South Korea, although the data from different studies could not be directly and statistically compared.

Frequent exposure to animals infected with HEV can lead to an increased risk of viral transmission in humans according to several reports showing that the HEV seroprevalence among swine veterinarians were higher than those of the general population [1, 5, 11, 12]. However, it remains unclear whether pet veterinarians are at higher risk of HEV transmission from companion animals based on the findings of the present and previous studies [10, 12]. Despite this limitation, pet animals and people who are in direct contact with infected animals must be continually monitored for public health concerns associated with HEV transmission.

In conclusion, HEV antibodies were detected in 28.2% of pet dogs in South Korea. While the sample size was relatively small, 5.0% of those tested were seropositive for pet veterinarians. The seropositivity rate among dogs with or without gastroenteritis symptoms was not significantly different. Although HEV seroprevalence in the general population was not examined in the present study, pet veterinarians are not at higher risk for HEV transmission. Therefore, our finding suggests that intensive monitoring of HEV infection and identification of HEV antigens in pet dogs is required to investigate the zoonotic potential of HEV transmission between humans and animals.

## Data Availability

The data that support the findings of this study are available from the corresponding author upon reasonable request.
